# Label-Free Visualization of the Antifungal Polyene Drug, Nystatin, in Biological Membranes Using Raman Microscopy

**DOI:** 10.1021/acs.analchem.5c07342

**Published:** 2026-05-04

**Authors:** William J. Tipping, Zainab Bilal, Robert C. Wells, Jason L. Brown, Duncan Graham, Karen Faulds

**Affiliations:** † Department of Pure and Applied Chemistry, 3527 University of Strathclyde, Technology and Innovation Centre, 99 George Street, Glasgow G1 1RD, U.K.; ‡ Oral Sciences Research Group, Glasgow Dental School, School of Medicine, College of Medical, Veterinary and Life Sciences, 3526 University of Glasgow, Glasgow G2 3JZ, U.K.; § Glasgow Biofilm Research Network, 378 Sauchiehall Street, Glasgow G2 3JZ, U.K.

## Abstract

Polyene macrolides are a class of antifungal drugs that remain essential for treating life-threatening fungal infections. The complex relationship between the chemical structure of the polyene macrolide, its interaction with biological membranes and subsequent intracellular localization, is poorly understood. Nystatin is an archetypal polyene macrolide, which has been in clinical use for the treatment of oral and dermal *Candida albicans* infections over several decades. To investigate the interaction of nystatin with biological membranes, we applied label-free stimulated Raman scattering (SRS) microscopy for this purpose. In mammalian cells, the formation of large extracellular aggregates of nystatin were observed, together with binding to the plasma membrane. In *C. albicans*, SRS imaging revealed a significant accumulation of nystatin in the fungal membrane and the internalization of the drug. Spectral phasor analysis was applied to identify the discrete nystatin signal from the cellular content using hyperspectral SRS imaging. This work provides the first label-free and direct visualization of nystatin binding in biological membranes and the subsequent trafficking in fungal cells using SRS microscopy.

## Graphical abstract

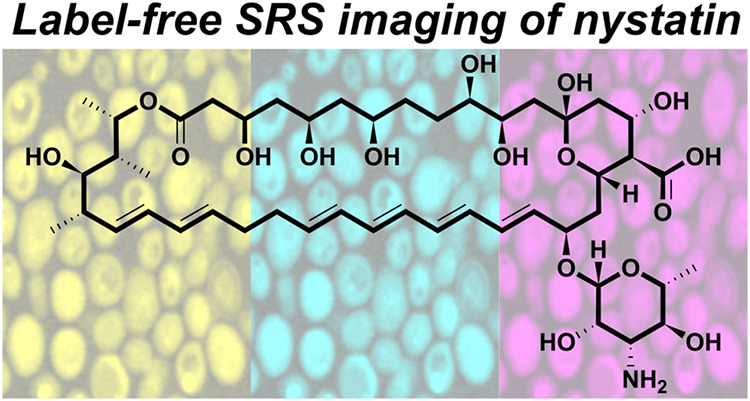

## Introduction

Nystatin is an antifungal polyene macrolide that is used for topical treatment of fungal infections.^1^ Unlike many other antifungal agents, resistance to nystatin is minimal in *Candida albicans*, and thus it remains a viable treatment option.^2^ Polyene macrolides are a class of antifungals that act by altering fungal cell membrane permeability through interaction with ergosterol, the primary sterol in fungi.^3,4^ The mechanism through which polyene macrolides exert a therapeutic response has not been fully delineated,^5^ although two models have been proposed to explain this interaction: the ion-channel model and the sterol sponge model.^6^

In the ion-channel model, aggregates of nystatin-ergosterol are proposed to form membrane pores, causing leakage of the intracellular contents and subsequent fungal cell death.^7–9^ In an alternative mechanism, referred to as the sterol sponge model, multimeric aggregates composed of polyene and ergosterol accumulate laterally on the surface of the fungal cell membrane, disrupting membrane integrity.^10^ The selectivity of nystatin for fungal cells compared to mammalian cells has been previously attributed to the higher affinity for ergosterol relative to cholesterol.^11,12^ However, the role of sterols in the mechanism of action and selectivity of nystatin and other polyene antifungal agents still remains challenging to study due to a lack of suitable methods to provide label-free, *in situ* detection.^13^

A variety of analytical methods have been used to study the interaction of polyene antifungal agents, including nystatin and amphotericin B (AmB), with biological membranes and models. To visualize the interaction of nystatin or AmB with cellular membranes, fluorescent tags have been appended to their molecular structures.^14^ For example, several fluorescently labeled nystatin and AmB analogues have been developed for fungal cell imaging (Figure S1).^15,16^ However, the perturbation of the binding of ergosterol by the modified polyene conjugates cannot be overlooked. Label-free imaging methods have thus attracted significant attention due to their unique ability to visualize molecular interactions in a native state. UV fluorescence has been used to study nystatin organization in Chinese Hamster Ovary (CHO) cells and *Saccharomyces cerevisiae* using intrinsic fluorescence from its conjugated tetraene structure.^17^ This study showed that nystatin aggregates formed on CHO cell membranes, which resulted in extensive ruffles and protrusions at the points of contact. However, the UV lasers used for imaging can be cell-damaging,^18^ and the detected membrane-bound signals were observed to be weak. Furthermore, interference from cellular autofluorescence is unavoidable in the region <400 nm.^19^

Label-free imaging of small molecules has been achieved using stimulated Raman scattering (SRS) microscopy.^20,21^ An advantage of SRS microscopy is that the use of near-infrared lasers for imaging is favorable for biological microscopy compared to UV laser excitation. As such, Raman-based techniques have facilitated a wide variety of applications in studying antimicrobial activity.^22^ Recently, SRS has enabled label-free visualization of AmB and its membrane organization in fungal cell models using the vibrational signal from the heptaene structure.^23^ Building on this work, we apply hyperspectral SRS imaging to investigate the binding and localization of nystatin in biological membranes to shed further insight into the mechanism of action of this important antifungal agent. We show that hyperspectral SRS imaging is capable of visualizing the membrane localization of nystatin together with the detection of nystatin aggregates at the cell periphery in mammalian cell culture. Our results demonstrate the potential of SRS microscopy for visualizing the membrane dynamics of nystatin in various biological models. In doing so, the interaction of this important class of antifungal compounds can be studied in a minimally invasive way.

## Experimental Section

### Chemicals and Reagents

Nystatin (92% purity) was received from Merck and prepared as a 10 mg/mL stock in DMSO. A fresh stock was prepared on the day of use. Ergosterol (pharmaceutical secondary standard) was received from Merck and used as supplied.

### Mammalian Cell Culture

PC-3 cells (CRL-1435) were received from Prof. Hing Leung (Beatson Institute, University of Glasgow) as a subculture from the American Type Culture Collection (ATCC) and were cultured in Dulbecco’s modified Eagle medium (DMEM, Gibco, Fisher Scientific) supplemented with 10% v/v fetal bovine serum (FBS, Gibco, Fisher Scientific) and 1% v/v penicillin/streptomycin (Gibco, 10,000 U mL^–1^, Fisher Scientific). The cells were routinely passaged at ca. 80% confluency.

### Mammalian Cell Imaging

PC-3 cells were plated at a density of 2.5 × 10^5^ cells/mL onto high precision glass coverslips (no. 1.5H thickness, 22 mm × 22 mm, Thorlabs) in a 6-well plate in regular medium. After 24 h, the cells were washed with PBS (3 mL × 2 mL) prior to culturing in DMEM containing the required concentration of nystatin. After the required treatment time had elapsed, the cells were mounted onto a glass microscope slide with a boundary of the culture medium (5 μL) and sealed with nail varnish. A minimum of three biological replicates were performed for each experimental condition.

### Fungal Cell Culture

*Candida albicans* SC5314 was stored on glycerol beads at −80 °C and recultured as required on Sabouraud agar (SAB, Oxoid). The streaked SAB plates were incubated aerobically at 30 °C for 48 h to allow the yeast to grow. Overnight cultures were prepared by inoculating 10 mL of yeast extract peptone dextrose (YPD, Sigma-Aldrich) with colonies from the streak plates and incubating at 30 °C for 24 h, with shaking at 180 rpm. Overnight cultures were centrifuged at 3000 rpm for 10 min in order to pellet the yeast cells, and the media was discarded. The pellet was washed twice with 10 mL of phosphate-buffered saline (PBS, Oxoid).

### Fungal Cell Imaging

The cells were pelleted using centrifugation (1000*g*), and the media was discarded. The pellet was resuspended in PBS (10 mL), vortex mixed, and centrifuged as described previously. The PBS supernatant was discarded, and the cell pellet was resuspended in PBS (500 μL) and treated with nystatin at the indicated concentration. The cell suspension was gently mixed prior to mounting onto a glass microscope slide (5 μL). A minimum of three biological repeats were performed for each experimental condition.

### Solution-Phase Nystatin Analysis

A 5 μL aliquot of nystatin solution prepared at the indicated concentration in PBS:DMSO (3:1 v/v) was pipetted onto a microscope slide. An appropriate quantity of nystatin was weighed before the addition of DMSO and then PBS to a final 3:1 *v/v* ratio solution was achieved. A coverslip was added to the slide and sealed with nail varnish. The SRS imaging parameters were equivalent to those used for mammalian and fungal cell imaging. SRS Spectra are plotted in Origin Pro. Peak fitting was performed in Origin Pro using the *Quick Peaks* function.

### Raman Spectroscopy

The Raman reference spectrum of nystatin was recorded using an inVia confocal Raman microscope (Renishaw) interfaced with a 785 nm diode laser (300 mW at source). The spectrum was acquired using a 5× objective (NA 0.12, Leica Microsystems) for 10 s exposure time, using 10% laser power (∼30 mW). The confocal Raman microscope was interfaced with a 1200 lines per millimeter grating and a CCD detector. Prior to spectral acquisition, the instrument was calibrated using an internal standard of Si at 520.5 cm^–1^. The spectrum is baseline corrected using a polynomial fitting (11th order) and plotted using Origin Pro.

### SRS Imaging

An integrated laser system (picoEmerald S, Applied Physics & Electronics, Inc.) was used to produce two synchronized laser beams at an 80 MHz repetition rate. A fundamental Stokes beam (1031.4 nm, 2 ps pulse width) was intensity modulated by an electro-optic modulator with >90% modulation depth, and a tunable pump beam (700–960 nm, 2 ps pulse width, <1 nm (10 cm^–1^) spectral bandwidth, polarization linear, horizontal >100:1) was produced by a built-in optical parametric oscillator. The pump and Stokes beams were spatially and temporally overlapped by using two dichroic mirrors and a delay stage inside the laser system and coupled into an inverted laser-scanning microscope (Leica TCS SP8, Leica Microsystems) with optimized near-IR throughput. SRS images were acquired using a 40× objective (HC PL IRAPO 40×, N.A. 1.10 water immersion lens). The Stokes beam was modulated with a 20 MHz EoM. Forward scattered light was collected by an S1 N.A. 1.4 condenser lens (Leica Microsystems). Images were acquired at a 12-bit image depth. The laser powers measured after the objective lens were ∼40 mW for the pump beam only, ∼70 mW for the Stokes beam only, and ∼100 mW (pump and Stokes beams). Hyperspectral SRS images were acquired across a 512 × 512 frame using a 9.75 μs/pixel dwell time and a 0.4 nm retuning of the pump beam in between image frames to create a data set of images across the range of 1690–1520 cm^–1^ (35 images). Under these conditions, the resulting frame rate was 0.097/s with a total acquisition of approximately ∼10 min for the hyperspectral stack. No baseline correction was applied to the spectra.

### Spectral Phasor Analysis

A plugin for ImageJ (FIJI) was used to perform spectral phasor analysis as described previously.^24^ Briefly, an average intensity projection was created from the hyperspectral SRS image stack, from which an intensity threshold was used to create a binary region-of-interest (ROI) image. The ROI was used to remove the background areas from the hyperspectral SRS image stack using the image calculator function available in FIJI prior to spectral phasor analysis. The real and imaginary components of the resulting first harmonic of the Fourier transform are used as positions in a scatter plot in the phasor space, as reported previously.^25^ The regions in the spectral phasor analysis were determined using manual segmentation of the phasor plot based on the resulting structures they produced and the localization pattern of the resultant phasor plot. Using the phasor analysis of nystatin in solid and solution phase (Figure 1) as a guide, the location of phasors relating to nystatin was determined, and an ROI marker at these coordinates was added to the ROI Manager on FIJI. Segmentation of the phasor plots for the PC-3 and *C. albicans* was performed using the specified ROI from the manager for consistency across data sets. The segmented images presented in the manuscript were false colored based on their localization pattern. The SRS spectra were used for determining the chemical composition of the segment. The spectra reflect the average spectrum from the whole stack of images, following background subtraction (i.e. removal of non-cell areas). The spectral phasor plots have been cropped for the sake of image clarity. The full phasor plots are available in the data file accompanying this manuscript. The corresponding SRS spectra were plotted using an Origin Pro.

**Figure 1:**
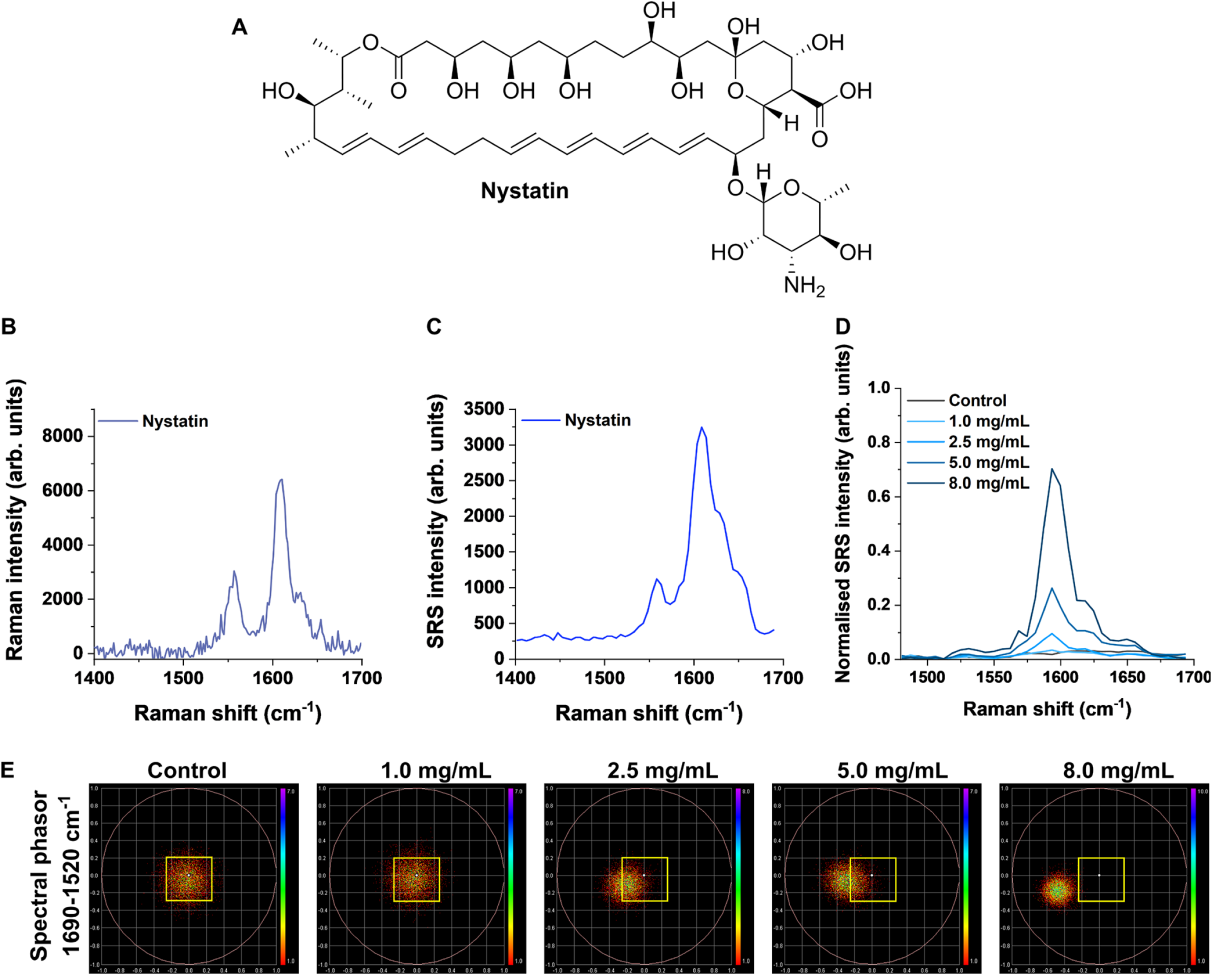
Analysis of nystatin using Raman spectroscopy. (A) Chemical structure of nystatin. (B) Raman spectrum of nystatin in solid form acquired using a 785 nm laser with a 5× lens (∼30 mW) for 10 s. An unprocessed spectrum is provided in Figure S2. (C) SRS spectrum of nystatin in solid form. (D) SRS spectra of nystatin in PBS:DMSO 3:1 *v/v*. The spectra are normalized to the intensity of the peak at 1419 cm^–1^ (DMSO). Please refer to the Materials and Methods for the acquisition parameters for (C, D). (E) Spectral phasor analysis of the nystatin solutions prepared in (D). A yellow ROI has been added to show the localization of the phasors in the control phasor plot.

## Results and Discussion

Nystatin has an amphipathic character due to the hydrophobic polyene structure and hydrophilic fucosamine and polyol moieties (Figure 1A). We first acquired the Raman and SRS spectra of nystatin in solid form to identify key peaks that could be used to enable its intracellular detection (Figure 1B,C).

The Raman stretching frequencies were in good agreement between the two modalities, particularly in the conjugated alkene (C=C) backbone region (1550–1650 cm^–1^). This result supports previous observations that the SRS spectrum closely resembles the Raman spectrum.^27^ We tabulated the spectral assignments for the solid nystatin in Table 1, which further showed good agreement between the spectral peak positions across the two analytical techniques. We also performed hyperspectral SRS imaging of nystatin in PBS:DMSO solution, which showed that at >2.5 mg/mL, the characteristic Raman peaks attributed to nystatin in the region 1550–1650 cm^–1^ were clearly observable (Figure 1D). However, at a reduced nystatin concentration (1 mg/mL), there was an undetectable difference relative to that of the PBS:DMSO solvent (Figure S2). The SRS images were acquired in series using a 0.4 nm retuning of the pump laser in between image frames. Thus, a three-dimensional (3D) hyperspectral data set was created such that each pixel location contained an SRS spectral profile. We elected to use spectral phasor analysis to analyze the 3D data set. Phasor analysis is a Fourier transform-based technique that projects each pixel in a 3D data set as a 2D representation in the phasor space.^28^ In doing so, phasors that are spatially close will share the same or similar spectral features. Conversely, phasors that are distant on the phasor plot will have markedly different overall spectral profiles. Spectral phasor analysis has been widely applied to hyperspectral SRS data for biological imaging applications, including cellular segmentation and classification,^25,29–31^ cytometry,^32^ exploring drug–cell interactions,^24,33^ and studying chemoresistance mechanisms.^34^ We performed spectral phasor analysis of the hyperspectral data for each nystatin solution (Figure 1E). As the concentration of nystatin increased, there was a corresponding shifting of the location of phasors in the plot away from the origin. The localization of the phasors at higher concentrations was consistent with the localization of phasors in the analysis of nystatin in solid form (Figure S3). With a defined localization pattern of nystatin under the hyperspectral SRS imaging settings used, we investigated the interaction of nystatin in biological models.

**Table 1:** Spectral Assignments for Nystatin^a^,^b^

Raman nystatin solid (cm^–1^)	SRS nystatin solid (cm^–1^)	nystatin aggregate (cm^–1^)	assignment^26^
1653	1651	1656	Asymmetric C=C stretch
1632	1629	1629	Diene C=C stretch
1607	1609	1609	Tetraene C=C stretch
1555	1558	1552	Heptaene C=C stretch

a Raman and SRS spectra are reported in Figure 1B–D.

b In nystatin, the detection of a Raman band at 1555 cm^–1^ (heptaene C=C stretch) has been attributed to an unknown impurity.

Nystatin has been shown to interfere with membrane function and integrity, particularly in assessing endocytic uptake pathways in mammalian cell culture (at ∼50 μg/mL), while at concentrations above 150 μg/mL, nystatin has been shown to induce cell blebbing and rupture.^35^ We therefore elected to investigate nystatin interaction in a live mammalian cell culture model in the first instance. To do so, we treated PC-3 cells (a model of prostate adenocarcinoma) with nystatin (50 μg/mL, 2 h) prior to imaging using SRS microscopy across the fingerprint region of the Raman spectrum (1690–1520 cm^–1^). We chose to use PC-3 cells as a suitable cellular model that has previously been characterized by hyperspectral SRS imaging in our earlier work.^36^

Spectral phasor analysis was performed on the hyperspectral images acquired from PC-3 cells treated with nystatin (50 μg/mL, 2 h) or DMSO as a control (Figure 2A). The resulting phasor plots were segmented into the following regions of interest: (i) cyan and (ii) yellow. A maximum intensity projection from the image stack is also provided in each case. The corresponding spectra for the two regions of interest, together with the mean SRS signal, are listed in Figure 2B–D. Nystatin was detected as a discrete signal in the cell boundary area, which we proposed to be membrane-bound, consistent with the ion-channel binding model.^10^ Importantly, we observed a bright signal for nystatin, which appeared to be aggregated on the periphery of most cells in the field-of-view (yellow arrows). The Raman shifts for the aggregate signals are also included in Table 1, which confirms the presence of nystatin. In addition, a line-plot analysis confirmed that the aggregated nystatin signal was bound to the external cell membrane (Figure 2E). The bright aggregates formed on the membrane periphery of the cells had a typical size in the range of 2.5–10 μm^2^ (Figure 2F). We also performed hyperspectral SRS imaging of nystatin in RPMI 1640 media to confirm the absence of precipitated signal (Figure S4). These observations are in close agreement with a previous report that showed the presence of nystatin aggregates bound to the membrane of CHO cells using UV fluorescence.^17^ Interestingly, using SRS microscopy, the detection of membrane-bound nystatin was observed around some cells together with the simultaneous detection of the membrane-bound aggregates. Whereas, in a previous report, the signal was shown to gradually decrease with increasing distance from the aggregate, due to the low intensity signal of the membrane-bound nystatin using UV fluorescence.^17^ Our results clearly show that nystatin binds to the membrane of mammalian cells not only in large aggregates but also surrounding the cell surface.

**Figure 2:**
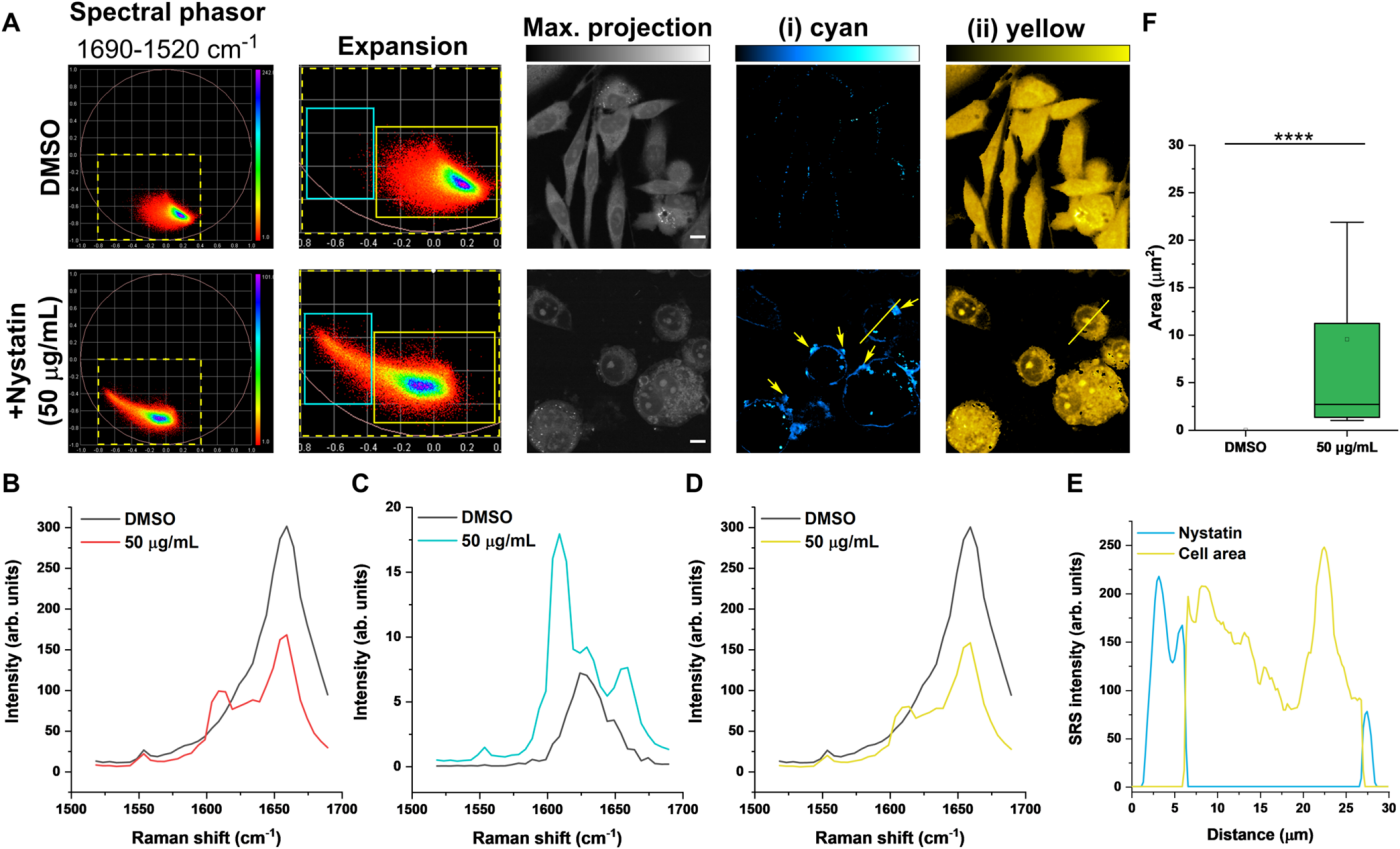
Hyperspectral SRS imaging of nystatin localization in PC-3 cells. (A) Live PC-3 cells were treated with DMSO (control) or nystatin (50 μg/mL, 2 h) before hyperspectral SRS imaging across the range 1690–1520 cm^–1^ (35 images, see Figure S5). Spectral phasor analysis was performed on the image stack. An expanded view of the phasor plot has been segmented into the following areas: (i) cyan ROI and (ii) yellow ROI. Yellow arrowheads are used to indicate nystatin aggregates. A maximum intensity projection is provided. Scale bars: 10 μm. (B) Mean SRS spectra for the control and nystatin-treated cells in (A). (C) Corresponding SRS spectra for the (i) cyan ROI in the control and nystatin-treated cells in (A). (D) Corresponding SRS spectra for the (ii) yellow ROI in the control and nystatin-treated cells in (A). (E) Intensity line-plot for the cell area and nystatin signals in the nystatin-treated cells in (A) (yellow line). (F) Quantification of the area of the aggregates detected in the (i) cyan ROI in (A). Data are collected from three biological repeats with a minimum feature size >0.5 μm^2^. Statistical significance was determined using a one-way ANOVA with a post-hoc Tukey test; ****P ≤ 0.0001.

We also showed that the presence of membrane-bound aggregates occurred rapidly when the concentration of nystatin was increased to >50 μg/mL (Figure 3). In these examples, we detected larger membrane-bound aggregates of nystatin at 100 and 200 μg/mL after a 15 min treatment time (Figure 3E). From the phasor analysis, it is also clear that the PC-3 cells presented a rounded morphology following treatment with higher nystatin concentrations. The hyperspectral SRS data from two ROIs and the mean SRS spectra are listed in Figure 3B–D. As the concentration of nystatin was increased, the SRS spectrum associated with the (i) cyan ROI (Figure 3C) became more similar to the SRS spectrum of solid nystatin (Figure 1C) and showed an increased trend in the overall intensity. From our imaging and spectroscopy experiments, it is not yet possible to determine if the nystatin aggregates that form on the plasma membrane of the cell recruit nystatin from the membrane or from the extracellular solution when they grow in size. However, it is clear that higher nystatin concentrations lead to larger aggregates that were detected on the extracellular membrane of the cells.

**Figure 3:**
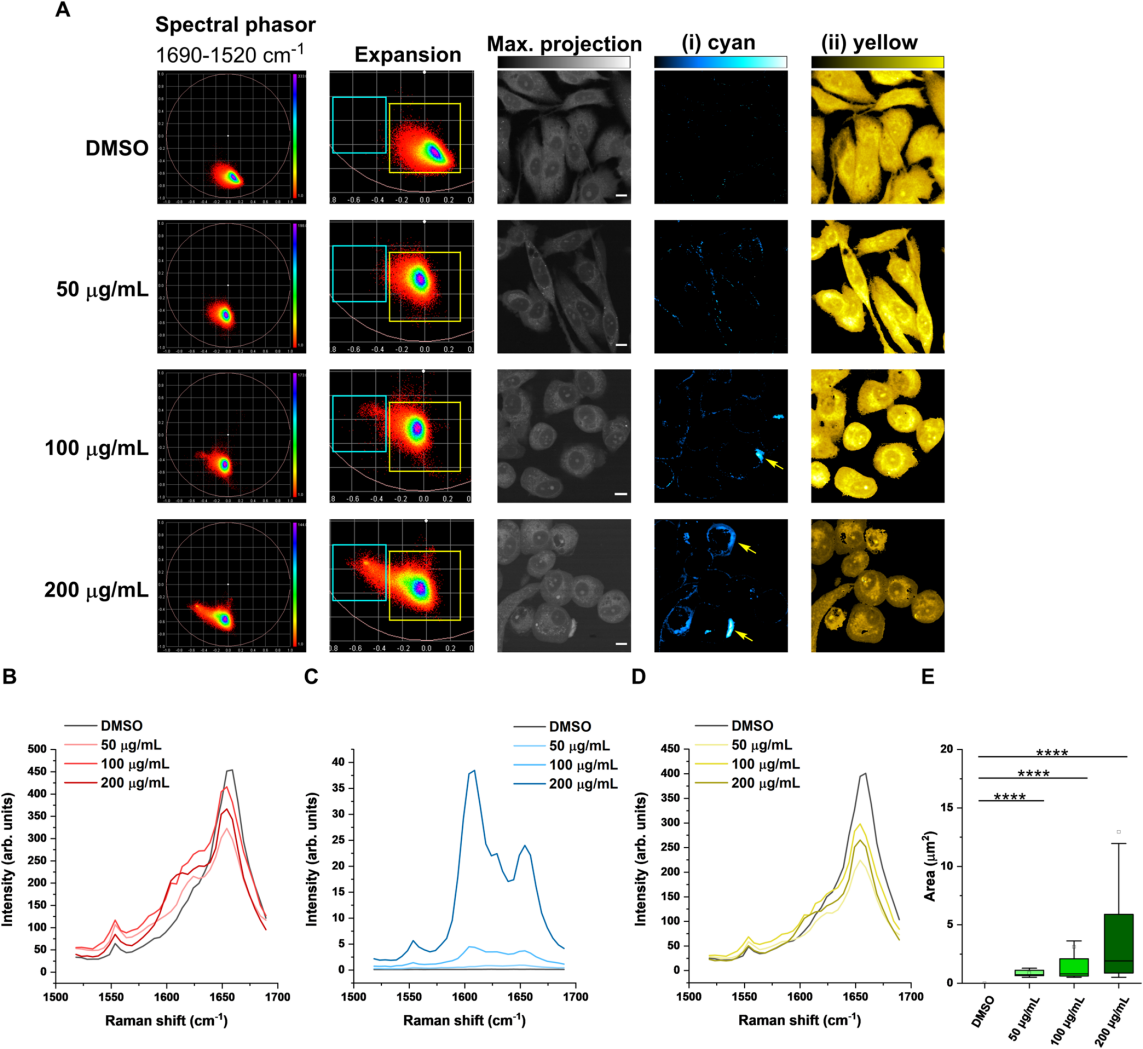
Investigating nystatin treatment concentration in mammalian cells. (A) PC-3 cells were treated with DMSO or nystatin at the indicated concentrations for 15 min prior to hyperspectral SRS imaging across the range 1690–1520 cm^–1^ (see Figure S5). Spectral phasor analysis was performed on the hyperspectral data set, which was segmented into the following regions: (i) cyan and (ii) yellow ROIs. Yellow arrowheads are used to identify large nystatin aggregates. A maximum intensity projection is provided. Scale bars: 10 μm. (B) Mean SRS spectra for the control and nystatin-treated cells in (A). (C) Corresponding SRS spectra for the (i) cyan ROI in the control and nystatin-treated cells in (A). (D) Corresponding SRS spectra for the (ii) yellow ROI in the control and nystatin-treated cells in (A). (E) Quantification of the area of the aggregates detected in the (i) cyan ROI for each condition in (A). Data are collected from three biological repeats with a minimum feature size >0.5 μm^2^. Statistical significance was determined using a one-way ANOVA with a post-hoc Tukey test; ****P ≤ 0.0001.

Nystatin is known to exert a toxic response in many fungal cells.^37^ To determine whether it is possible to visualize nystatin in fungal cell membranes, we incubated *C. albicans* with 50 or 100 μg/mL nystatin in nutrient-free PBS buffer (pH 7.4) for 2 h, followed by SRS microscopy (Figure 4). A control population was treated with an equivalent volume of DMSO. Hyperspectral SRS imaging across the fingerprint region was performed prior to spectral phasor analysis. Our results showed that in nystatin-treated cells, two main regions of phasor clustering were observed, labeled (i) cyan and (ii) yellow, while only one region was evidenced in the control ((ii) yellow (dashed)). Interestingly, the control-treated cells were in a cluster that was localized away from the yellow ROI detected in the nystatin-treated cells. Segmentation of the phasor plots revealed large areas of aggregated nystatin signal in the extracellular space, together with some bright signals in the membrane of some, but not all, *C. albicans* cells. The yellow ROI revealed the cell body of the fungal cells in both the control and nystatin-treated fungal populations. Interestingly, in the nystatin-treated *C. albicans*, the yellow ROI contained a significant SRS signal for intracellular nystatin at ∼1610 cm^–1^, which was notably absent in the DMSO control population (Figure 4B–D), which likely explains the difference in localization of the phasors between the control and nystatin-treated populations. In the nystatin-treated *C. albicans*, nystatin was detected as bright aggregates in the extracellular space (i) cyan ROI, together with strong plasma membrane staining and some weak intracellular nystatin signal, which was likely to be the vacuole membrane, as shown in (ii) yellow ROI. This distribution is consistent with that of a recently reported fluorescently tagged nystatin analogue (Figure S1).^38^ The intracellular accumulation of nystatin at 50 μg/mL is consistent with a stress-induced trafficking response and is likely to reflect the fact that, while the treatment time is short, the concentration is above the minimum inhibitory concentration (MIC) for nystatin in sensitive *C. albicans*.^39^ We included the hyperspectral images for the DMSO control (Figure S6) and *C. albicans* treated with 50 μg/mL (Figure S7) or 100 μg/mL (Figure S8) to highlight the detection of nystatin in the fungal cell membrane and intracellular regions at 1610 cm^–1^.

**Figure 4:**
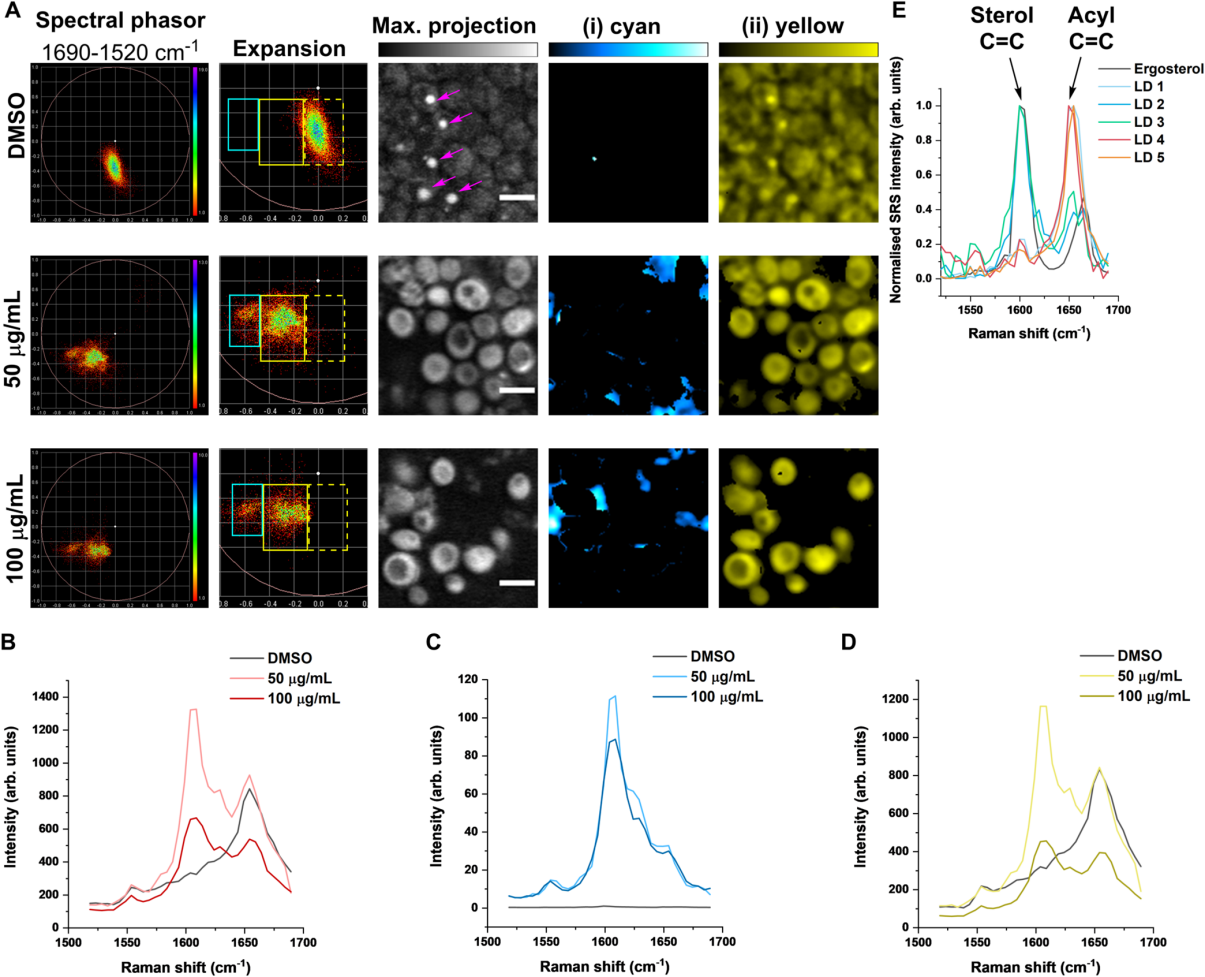
Subcellular distribution of nystatin in live *C. albicans* using hyperspectral SRS imaging. (A) Live *C. albicans* were treated with DMSO (control) or nystatin (50 or 100 μg/mL) in PBS for 2 h before SRS imaging across the range 1690–1520 cm^–1^. Spectral phasor analysis was performed on the hyperspectral data, which was segmented into two regions of interest: yellow ROI (cell body) and cyan ROI (nystatin). An average intensity projection is provided for each condition. In the DMSO control cells, magenta arrowheads are used to locate lipid droplets analyzed in (E). Scale bars: 5 μm. (B) Mean SRS spectra for the control and nystatin-treated cells in (A). (C) Corresponding SRS spectra for the (i) cyan ROI in the control and nystatin-treated cells in (A). (D) Corresponding SRS spectra for the (ii) yellow ROI in the control and nystatin-treated cells in (A). (E) SRS spectra of ergosterol (solid) and selected lipid droplets (LDs) from DMSO-treated *C. albicans* in (A).

The normalized SRS spectra showed that a significant nystatin signal was detected in the treated cells at both 50 and 100 μg/mL. Interestingly, we observed an intracellular nystatin signal in *C. albicans*, whereas in the mammalian cells, the nystatin signal was largely concentrated in the membrane region and in large extracellular aggregates. Thus, nystatin appears to bind more extensively and is internalized more effectively in *C. albicans* than in mammalian cells, which is commensurate with its selective antifungal properties. The observed selective internalization of nystatin could be attributed to the presence of ergosterol in the fungal cells. At 200 μg/mL, the viability of *C. albicans* was severely impaired, and hence, we omitted this concentration from our analysis.

Hyperspectral SRS imaging also revealed that many of the control-treated *C. albicans* contained a large unilocular lipid droplet, which is typical of this cell type (magenta arrowheads, Figure 4A).^40^ However, in the nystatin-treated cells, we did not observe lipid droplets in any of the cells investigated. We compared the SRS spectra of the lipid droplets present in the control-treated cells (Figure 4A) to the SRS spectrum of ergosterol in solid form (Figure 4E). Our analysis showed that most of the lipid droplets contained ergosterol, and some to a high level, as evidenced by the sterol C=C Raman band at 1605 cm^–1^ (Figure S9).^41^ We propose that in the nystatin-treated cells, the loss of the intracellular lipid droplets could be a result of ergosterol binding to nystatin, therefore resulting in an absence of large lipid droplets in these cells. Thus, ergosterol stored in *C. albicans* is directly impacted by nystatin treatment.

By comparing the hyperspectral SRS imaging on standard solutions of nystatin in DMSO (Figure 1D) with the hyperspectral SRS imaging in PC-3 cells (Figures 2 and 3) and *C. albicans* (Figure 4), it is possible to investigate the impact of nystatin aggregation in culture medium. At 10 mg/mL, we observed a strong SRS signal indicative of nystatin under the same imaging settings as used for biological imaging, while at 50 μg/mL, the treatment concentration in PC-3 and *C. albicans*, the SRS signal was indistinguishable from that of the solution. This result suggests that nystatin is enriched following treatment in mammalian and fungal cell membranes, both in membrane binding and in the formation of the aggregates detected. This result indicated that significant accumulation and aggregation of nystatin occurred in the culture medium for both species. The accumulation of drug molecules has been widely observed for different classes of drugs, including several tyrosine kinase inhibitors and bioactive natural products.^42,43^ This report shows that intracellular accumulation and aggregation of nystatin are important features of the action of this antifungal agent in biological models.

It is important to note that *C. albicans* exists as a biofilm in clinical infections, and the efficacy of nystatin will likely be different in a biofilm environment relative to planktonic cells. *C. albicans* is distinguished by its ability to undergo morphological switching, which is observed at different stages of biofilm formation.^44^ During the initial stages of biofilm development, *C. albicans* yeast cells will elongate to form pseudohyphae following budding and begin to form chains. Pseudohyphae may vary in size and length but are characterized by constrictions at the septal junctions. As the biofilm continues to grow and mature, the pseudohyphae elongate into long filaments known as true hyphae, which possess smooth cell walls and form large hyphal networks.^45^ True hyphae play several roles in establishing infection, such as increased adhesion, tissue invasion, and penetration of the host’s bloodstream. Combined with the secretion of the biofilm extracellular matrix, the development of this complex structure plays an essential role in preventing penetration of antimicrobials.^46^

In our study, we observed rapid accumulation of nystatin aggregates in culture medium at concentrations greater than 50 μg/mL. In order to preserve the integrity of the *C. albicans* used in this study, we elected to use relatively short incubation times. The treatment conditions used in our study (>50 μg/mL) are similar to the treatment concentrations used in other studies,^17^ and as observed by us and others, this concentration is likely to be greater than the minimum inhibitory concentration of nystatin in *C albicans*. We propose that nystatin activity be assessed at lower concentrations in live *C. albicans*. A suitable perfusion culture system or microfluidic system would be required that can deliver a lower treatment concentration over a longer duration. This is an area of active development^47^ that would enable the assessment of nystatin and other antifungal agents in living systems with longitudinal analysis. Overall, our results in mammalian and fungal cell models indicated a multifaceted interaction of nystatin with biological membranes. We detected the formation of nystatin aggregates on the cell membrane, which is consistent with the sterol sponge model of nystatin activity. In addition, our results showed clear membrane-bound signals in both cell systems, indicating the potential for both models of nystatin interaction to be implicated in its biological activity.

## Conclusions

This study presents the first example of direct subcellular imaging of nystatin in biological systems by using hyperspectral SRS imaging. The label-free detection of nystatin using the SRS spectrum to do so has enabled the visualization of nystatin aggregates in a dose-dependent manner in mammalian cells and *C. albicans*. Spectral phasor analysis enabled the discrimination of extracellular nystatin aggregates from the intracellular nystatin content in *C. albicans*, indicating the likelihood of both the ion-channel model and sterol sponge methods as potential mechanisms by which nystatin exerts a therapeutic effect. The development of a microfluidic platform to deliver reduced concentrations of nystatin at longer time points is required in order to assess nystatin activity at concentrations below the MIC, and with temporal resolution. Under our current imaging setup, it is not possible to ascertain if nystatin binds to the membrane before the formation of the extracellular aggregate signal. Nevertheless, this study establishes an analytical approach using hyperspectral SRS microscopy to investigate nystatin function and localization in live biological models, with the potential to investigate alternative polyene antifungal agents in various pathogenic fungi species.

## Supplementary Material



## Data Availability

The raw data corresponding to this manuscript will become available at the following link: 10.15129/1808e375-c2ad-43ac-a21e-b6ad8f8274f0.

## Notes

An electronic Supporting Information file is included with this manuscript, containing additional experimental details, materials, and methods, and Figures S1–S9; control SRS images and hyperspectral image stacks associated with the manuscript figures (PDF)

## Abbreviations

SRS: stimulated Raman scattering
ROI: region-of-interest
MIC: minimum inhibitory concentration
