# Supplementary material for: Label-Free Visualization of the Antifungal Polyene Drug, Nystatin, in Biological Membranes Using Raman Microscopy
Source: Anal Chem. 2026 May 4;98(19):14039–48. doi: 10.1021/acs.analchem.5c07342 (PMC13191723; doi:10.1021/acs.analchem.5c07342)
Supplement: Supplementary file 1 [file ac5c07342_si_001.pdf]

*Electronic Supplementary Information*

# Label-free visualisation of the antifungal polyene drug, nystatin, in biological membranes using Raman microscopy

William J. Tipping,[a],* Zainab Bilal,[a,b, c] Robert C. Wells,[a] Jason L. Brown,[b,c] Duncan Graham[a],* and Karen Faulds[a],*

[a] Department of Pure and Applied Chemistry, University of Strathclyde, Technology and Innovation Centre, 99 George Street, Glasgow, G1 1RD, UK.

Email: william.tipping@strath.ac.uk; duncan.graham@strath.ac.uk; karen.faulds@strath.ac.uk

[b] Oral Sciences Research Group, Glasgow Dental School, School of Medicine, College of Medical, Veterinary and Life Sciences, University of Glasgow, Glasgow, G2 3JZ, UK.

[c] Glasgow Biofilm Research Network, 378 Sauchiehall Street, Glasgow, G2 3JZ, UK.

**Contents**

**Supplementary Materials and Methods**......2

**Figure S1**......2

**Figure S2**......3

**Figure S3**......4

**Figure S4**......5

**Figure S5**......6

**Figure S6**......7

**Figure S7**......8

**Figure S8**......9

**Figure S9**......10

**References**......11

**Materials and Methods**

**SRS imaging** A integrated laser system (picoEmerald S, Ap-plied Physics & Electronics, Inc.) was used to produce two synchronized laser beams at a 80 MHz repetition rate. A fundamental Stokes beam (1031.4 nm, 2 ps pulse width) was intensity modulated by an electro-optic modulator with >90% modulation depth, and a tunable pump beam (700–960 nm, 2 ps pulse width, <1 nm (10 cm$^{-1}$) spectral bandwidth) was produced by a built-in optical parametric oscillator. The pump and Stokes beams were spatially and temporally overlapped using two dichroic mirrors and a delay stage inside the laser system and coupled into an inverted laser-scanning micro-scope (Leica TCS SP8, Leica Microsystems) with optimized near-IR throughput. SRS images were acquired using a 40× objective (HC PL IRAPO 40×, N.A. 1.10 water immersion lens). The Stokes beam was modulated with a 20 MHz EoM. Forward scattered light was collected by an S1 N.A. 1.4 condenser lens (Leica Microsystems). Images were acquired at a 12-bit image depth. The laser powers measured after the objective lens was ~40 mW for the pump beam only, ~70 mW for the Stokes beam only, and ~100 mW (pump and Stokes beams). Hyperspectral SRS images were acquired across a 512 × 512 frame using a 9.75 µs/pixel dwell time and a 0.4 nm retuning of the pump beam in between image frames to create a data set of images across the range 1690-1520 cm$^{-1}$ (35 images). Under these conditions, the resulting frame rate was 0.097/s with a total acquisition of approximately ~10 min for the hyperspectral stack. A set of three biological replicates were performed for each experimental condition.

**Ergosterol imaging:** a µg quantity of ergosterol solid was placed onto a clean microscope slide and a coverslip added on top. The coverslip was sealed with nail varnish before imaging. SRS images were acquired across the region 1690-1520 cm$^{-1}$ using the same settings as the *C. albicans* imaging. A small ROI (38 µm × 38 µm) was selected from the image stack and presented in **Figure S9**.

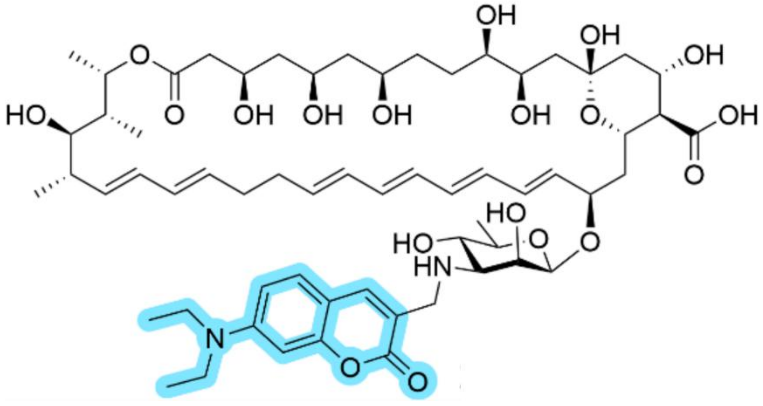


**Figure S1** Molecular structure of a recently reported fluorescent nystatin analogue. Structure reproduced from Ref. 1.

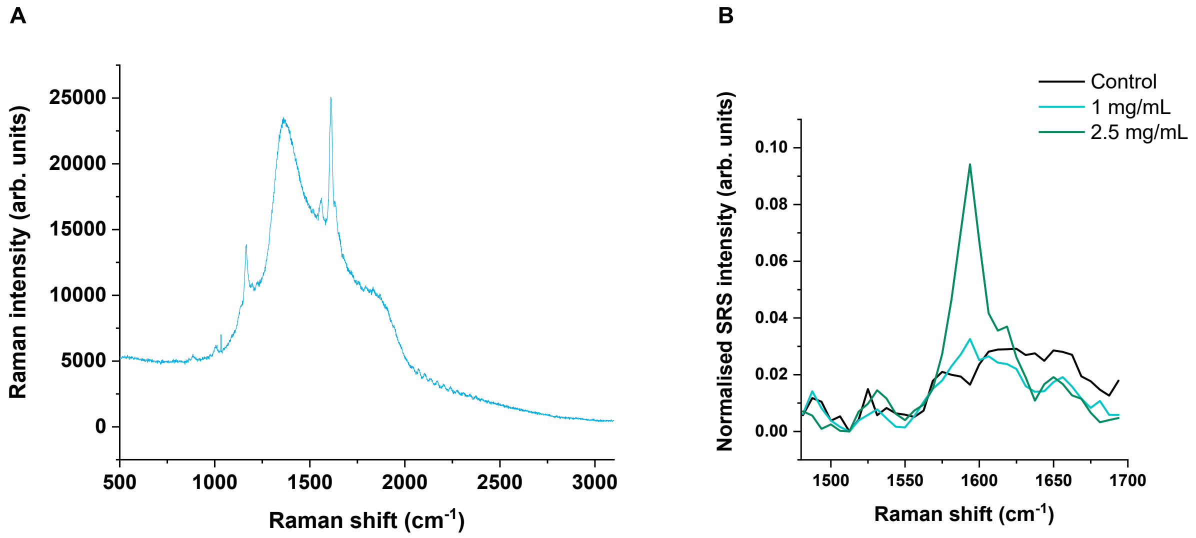


**Figure S2** Additional materials associated with Figure 1. **A** Unprocessed Raman spectrum corresponding to Figure 1B. **B** Re-scaled SRS spectra corresponding to Figure 1D. At 1.0 mg/mL, the spectrum is indistinguishable from the control, whereas the 2.5 mg/mL solution spectrum clearly shows a peak indicative of nystatin at ~1600 $cm^{-1}$. Please refer to Figure 1 for acquisition parameters in both cases.

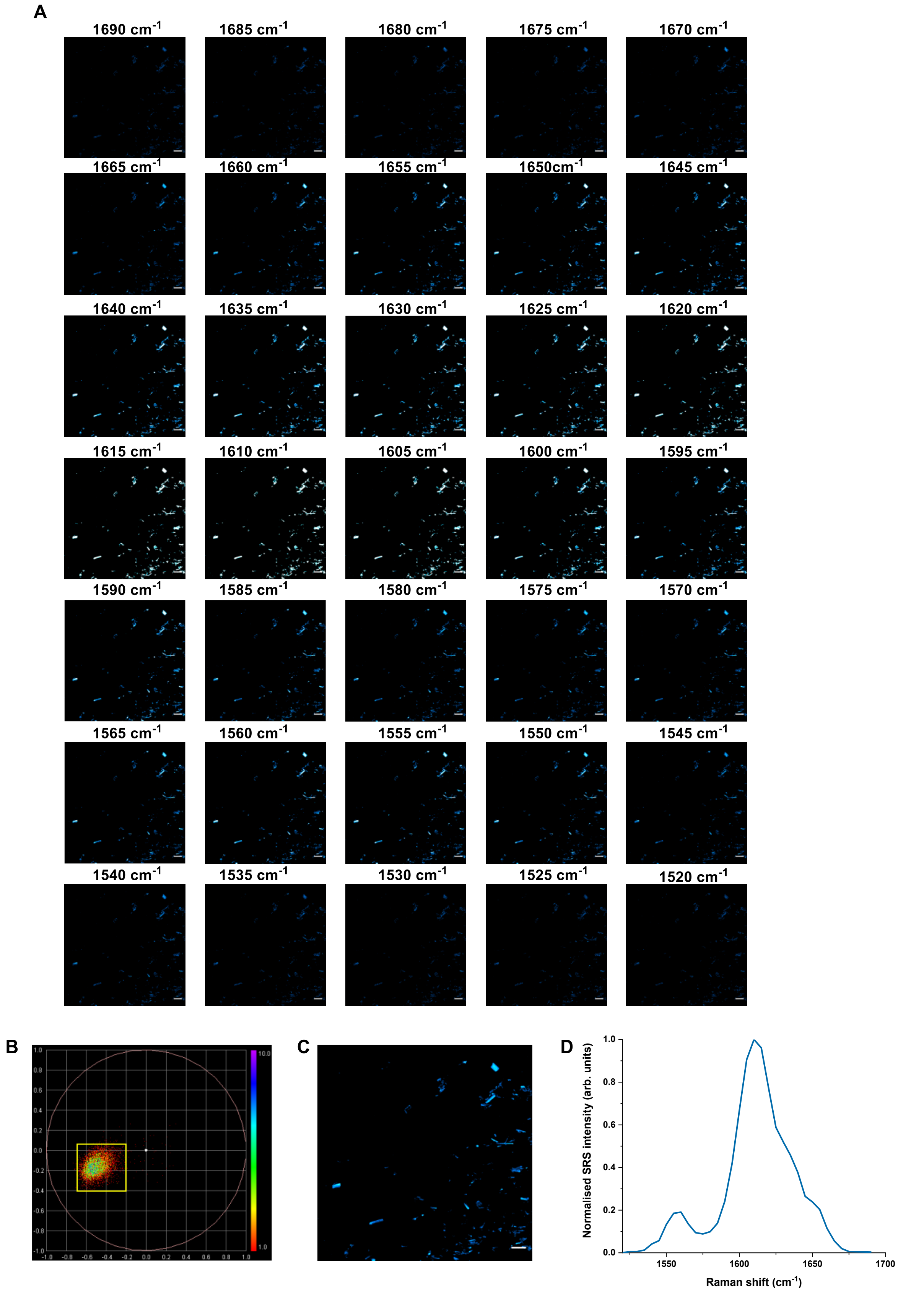


**Figure S3** Hyperspectral SRS imaging of nystatin in solid form. **A** Hyperspectral images were acquired across the range 1690-1520 $cm^{-1}$ by re-tuning of the pump beam at 0.4 nm increments in between image frames. SRS images presented in cyan Hot LUT. The background signals have

been removed (see Materials and Methods). The images are scaled 0-4095 intensity units. Each image represents a frame size of 291.19×291.19 µm. Scale bars: 20 µm. **B** Spectral phasor analysis of the hyperspectral SRS images presented in **A**. The yellow ROI marker highlights the main clustering of phasors associated with nystatin in solid form. **C** Segmented phasor image from the region identified by the yellow ROI in **B**. **D** The normalised SRS spectrum for the phasors selected in the yellow ROI in **B**.

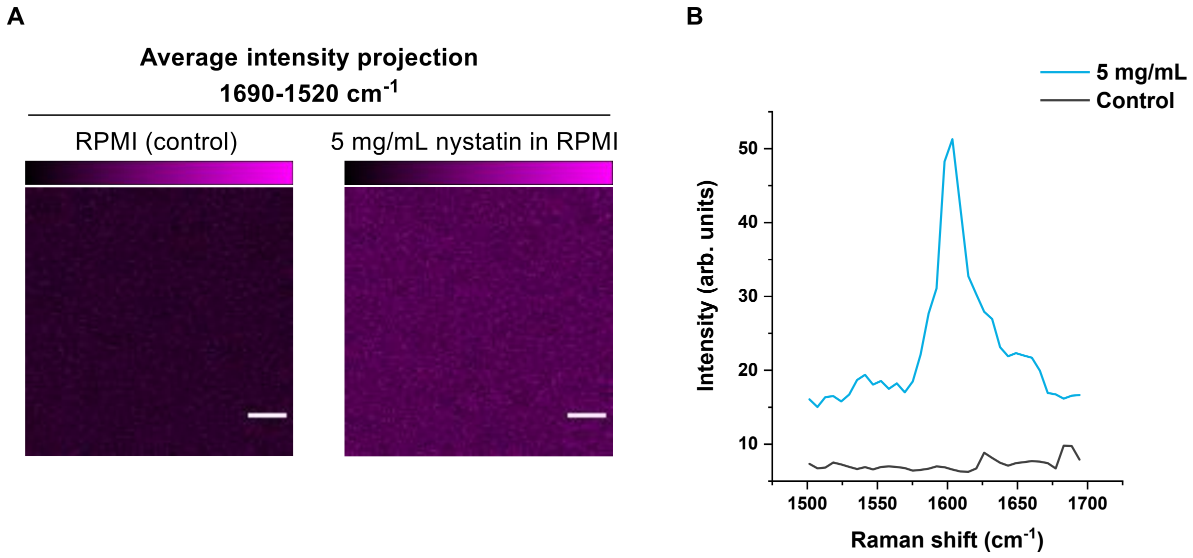


**Figure S4** Analysis of nystatin in RPMI media. **A** SRS images were acquired across the range 1690-1520 $cm^{-1}$ from a solution of RPMI (control) or nystatin (5 mg/mL in RPMI). An average intensity projection shows (i) an absence of precipitated nystatin (uniform signal) and (ii) the average intensity projection is more intense for the solution containing nystatin than the control. The images are presented in a magenta LUT and scaled 0-4095 intensity units. Scale bars: 10 µm. **B** Average SRS spectra from the two conditions analysed in **A**.

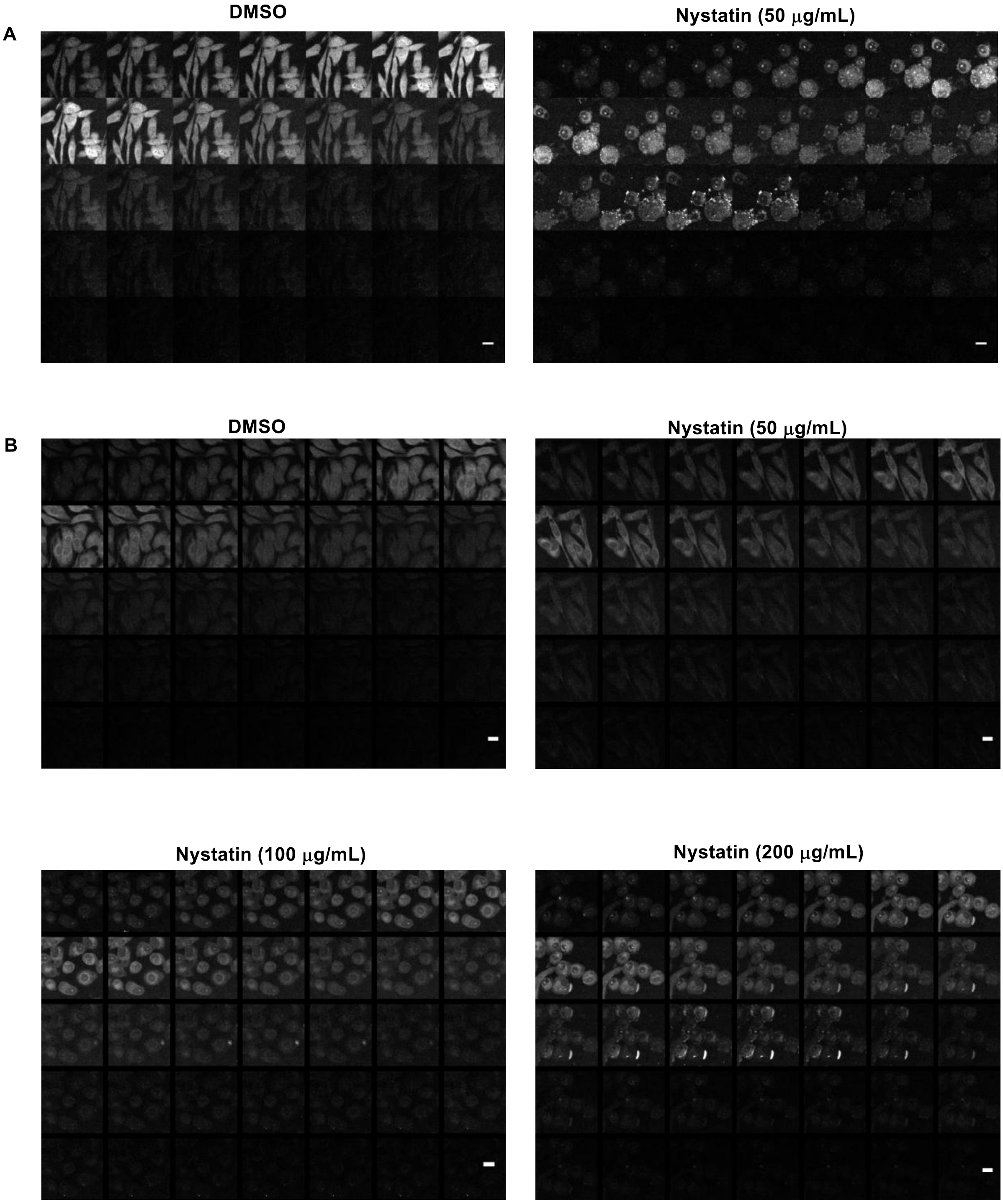


**Figure S5** Hyperspectral SRS imaging of PC-3 cells. **A** Corresponding SRS spectral image stacks across the range 1690-1520 $cm^{-1}$ from Figure 2. Images presented in greyscale LUT and scaled 0-1000 digitiser counts. **B** Corresponding SRS spectral image stacks across the range 1690-1520 $cm^{-1}$ from Figure 3. Images are unprocessed, presented in greyscale LUT and scaled 0-1000 digitiser counts. Scale bars: 20 μm.

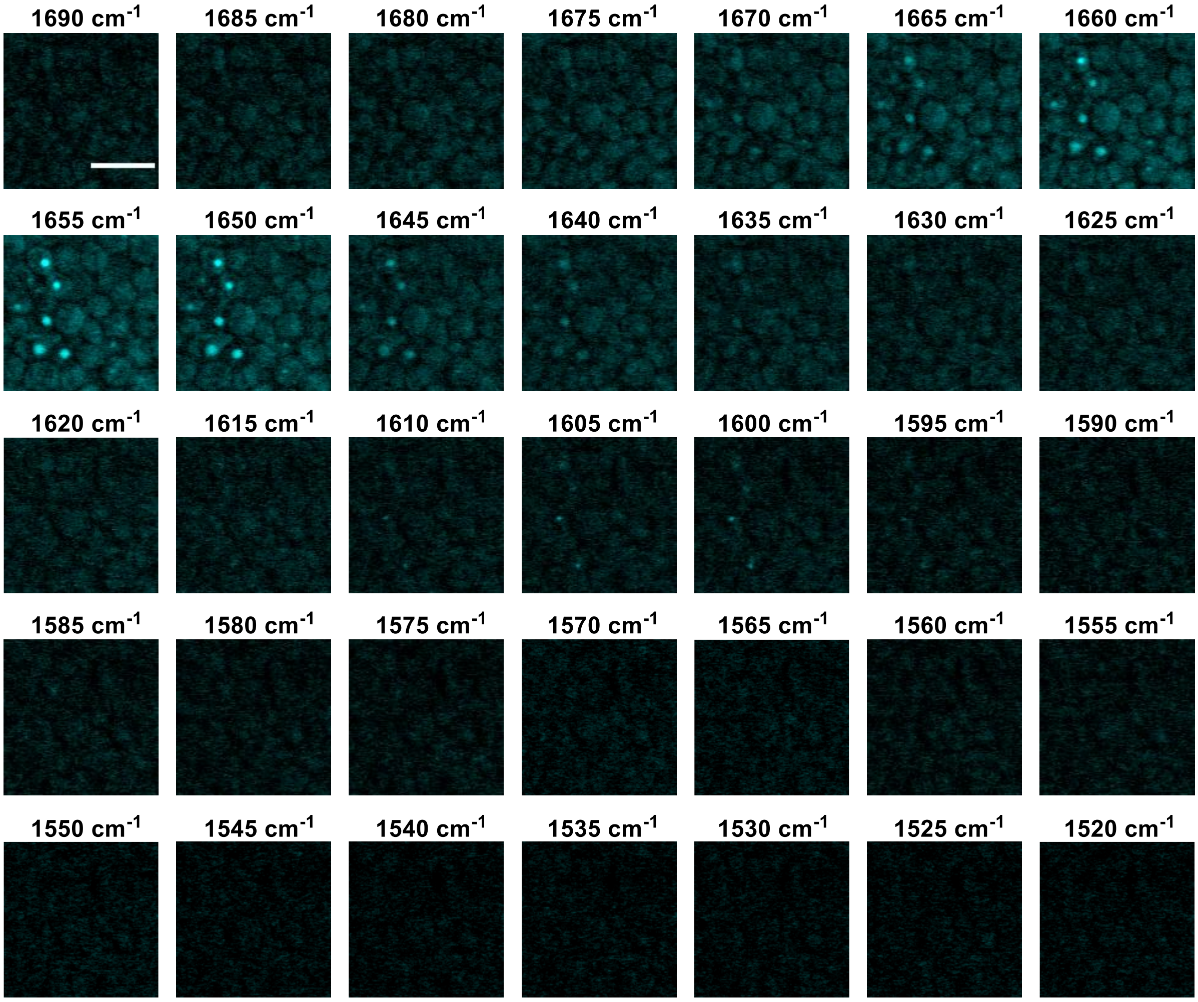


**Figure S6** Hyperspectral SRS imaging of live *C. albicans* in PBS. A control population of *C. albicans* were imaged by hyperspectral SRS imaging across the region 1690-1520 $cm^{-1}$ using a 5 $cm^{-1}$ retuning of the pump beam wavelength. The images are unprocessed, presented in a cyan look-up table and are scaled 0-4095 intensity units. Scale bar: 10 µm.

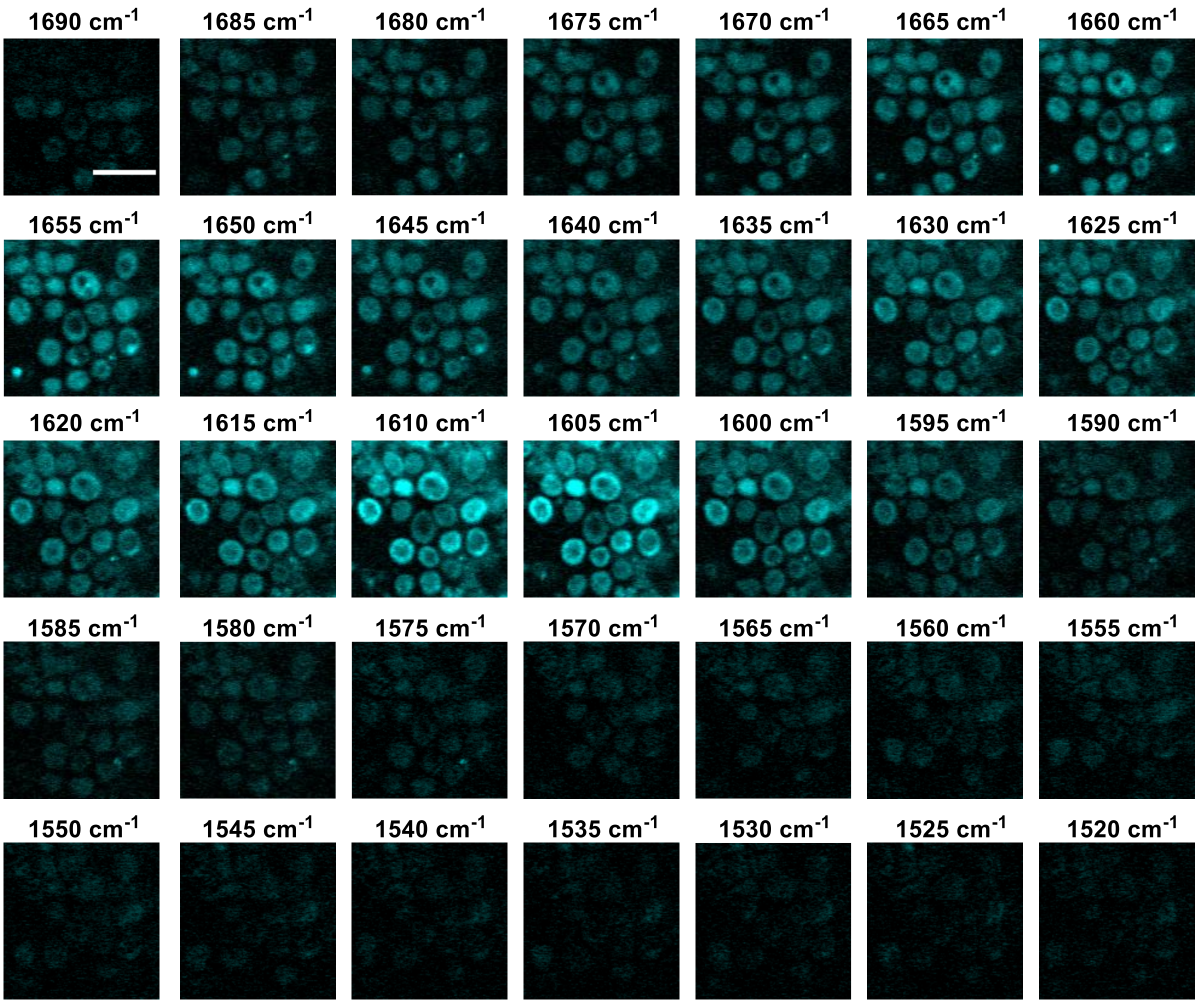


**Figure S7** Hyperspectral SRS imaging of live *C. albicans* in PBS. A population of *C. albicans* were treated with nystatin (50 µg/mL in PBS) before being imaged by hyperspectral SRS imaging across the region 1690-1520 $cm^{-1}$ using a 5 $cm^{-1}$ retuning of the pump beam wavelength. The images are unprocessed, presented in a cyan look-up table and are scaled 0-4095 intensity units. Scale bar: 10 µm.

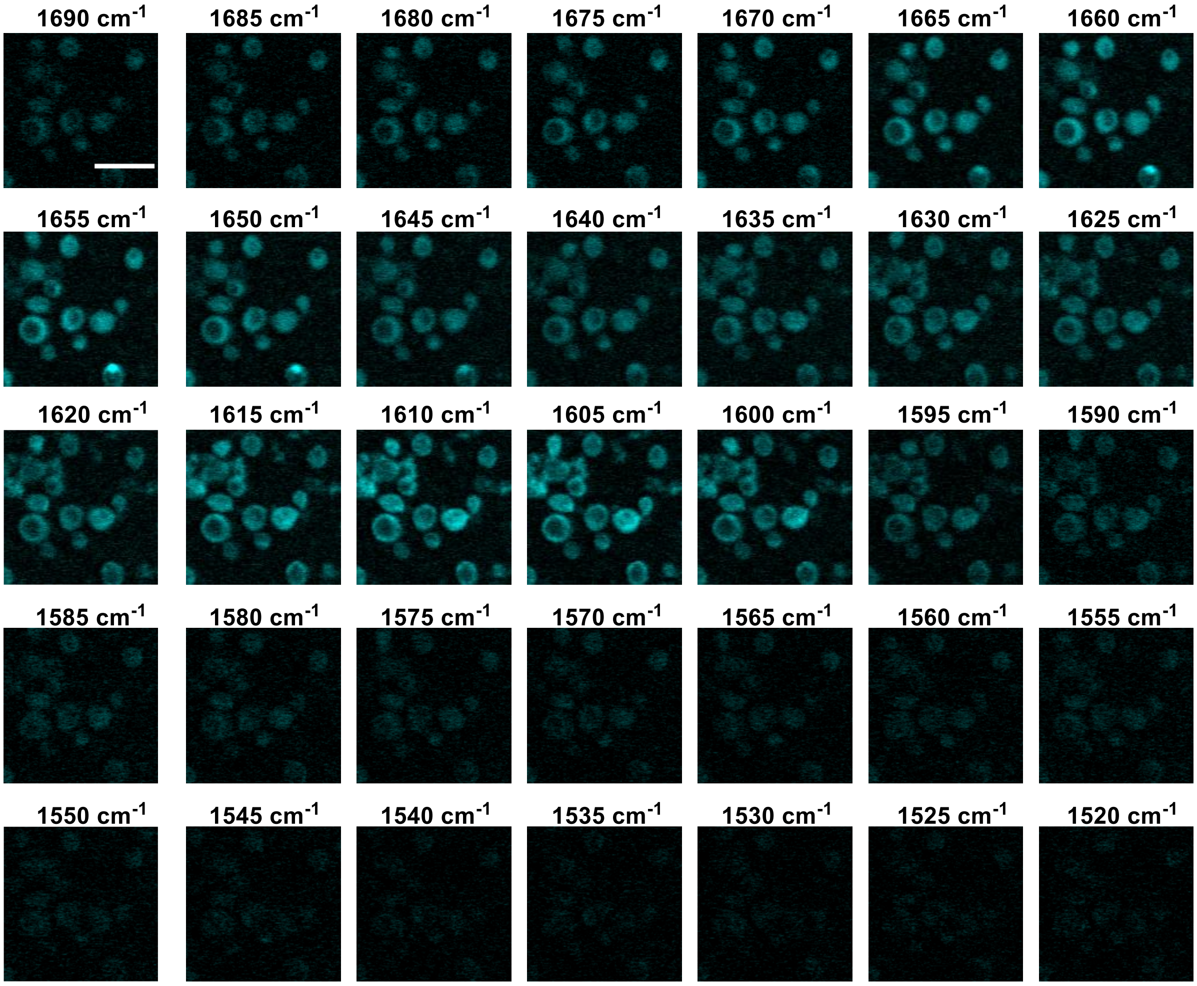


**Figure S8** Hyperspectral SRS imaging of live *C. albicans* in PBS. A population of *C. albicans* were treated with nystatin (100 µg/mL in PBS) before being imaged by hyperspectral SRS imaging across the region 1690-1520 $cm^{-1}$ using a 5 $cm^{-1}$ retuning of the pump beam wavelength. The images are presented unprocessed, presented in a cyan look-up table and are scaled 0-4095 intensity units. Scale bar: 10 µm.

When compared to the DMSO control, a significant SRS signal is detected at 1610 $cm^{-1}$ indicative of nystatin. In some cells, we observed a strong signal emanating from the cell membrane, whereas in others, this signal is also detected in the intracellular compartment. The dual localisation pattern is consistent with that observed for a fluorescent nystatin analogue (**Figure S1**).[1]

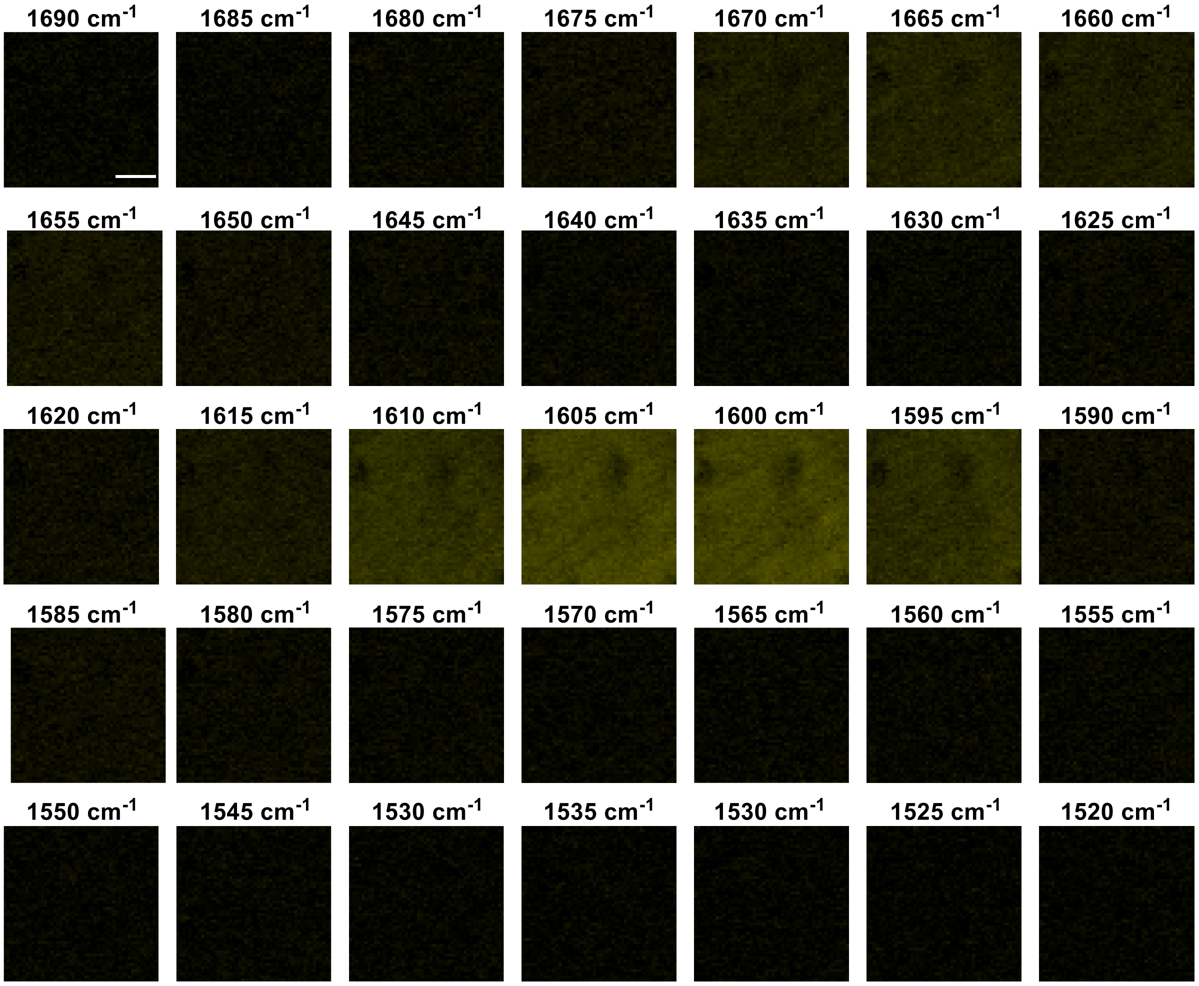


**Figure S9** Hyperspectral SRS imaging of ergosterol in solid form. Hyperspectral SRS imaging across the region 1690-1520 $cm^{-1}$ using a 5 $cm^{-1}$ retuning of the pump beam wavelength. The images are unprocessed, presented in a yellow look-up table and are scaled 0-4095 intensity units. Scale bar: 10 μm.

**References**

[1] M. Shbeta, T. Kopp, I. Voronov, A. Yona, R. Hasib Afana, S. Carmeli and M. Fridman. **Fluorescent Probes Derived from the Polyene Class of Antifungal Drugs Reveal Distinct Localization Patterns and Resistance-Associated Vacuolar Sequestration in *Candida* Species.** *Angew Chem Int Ed,* **2026,** 138**,** e21135**.**
